# Mandibular symmetry on posterior-anterior cephalograms of neurofibromatosis type 1 patients with facial plexiform neurofibroma

**DOI:** 10.3205/iprs000181

**Published:** 2023-12-11

**Authors:** Reinhard E. Friedrich, Georg Christ, Hanna A. Scheuer

**Affiliations:** 1 Oral and Craniomaxillofacial Surgery, Eppendorf University Hospital, University of Hamburg, Germany; 2 Department of Orthodontics, Eppendorf University Hospital, University of Hamburg, Germany; 3 Private Praxis of Orthodontics, Lokstedt, Hamburg, Germany

**Keywords:** neurofibromatosis type 1, plexiform neurofibroma, mandible, cephalometry, symmetry, bone, facial skeleton

## Abstract

**Introduction:**

Neurofibromatosis type 1 (NF1) is an is an autosomal dominant heritable tumor predisposition syndrome.. Peripheral nerve sheath tumors (PNST) are a hallmark of NF1. Plexiform neurofibromas (PNF) are neoplasms that are characteristic of NF1, often causing disfiguring effects (e.g., on the face), and are considered precancerous lesions. Previous studies have shown that facial PNF (FPNF) have an impact on the shape of facial bones. This study examines deviations of mandibular symmetry from cephalometric reference planes considering the topography of FPNF.

**Material and methods:**

The posterior-anterior (PA) cephalograms of 168 patients with NF1 were examined. We compared three groups: patients with FPNF (n=74), with disseminated cutaneous neurofibroma (DNF (n=94)), and control subjects without NF1 (n=23). The PNF group was subtyped with respect to facial PNST type and location. Typical mandibular cephalometric reference points were determined (condyle, antegonion, and menton).

**Results:**

The skeletal measurement points of the mandible in FPNF patients often differ significantly from those of the DNF group. It has been proven that typical asymmetries of the median-sagittal measurement points are indicators of PNF. Differences within the trigeminal tumor spread patterns are indicated in the measured values. A local tumor effect (PNF) on the relation of the measurement points to the reference planes is made plausible by the study results. The investigations prove that tumor type (FPNF) and the number of FPNF affected branches of the trigeminal nerve may correlate with significant deviations of mandible from symmetry on PA projections.

**Conclusion:**

The presented study shows that characteristic patterns of mandibular deformity can be measured on standardized radiographs in NF1 patients with FPNF. Mandibular deformities imaged on standardized radiographs may be initial indicators of a previously unrecognized NF1. Tumor-associated alterations of the mandible should be considered in the classification systems of pathognomonic, diagnostically pioneering osseous findings in NF1. The radiological findings provide clues for planning mandibular osteotomies in NF1 patients, especially for assessing facial regions typically highly vascularized by tumor spread. Furthermore, the radiological findings are an indication of a tumor potentially invading and destroying adjacent masticatory and mimic muscle, findings that may have an influence on surgical measures (function, aesthetics, and wound healing).

## Zusammenfassung

**Einleitung:**

Neurofibromatose Typ 1 (NF1) ist ein autosomal dominant vererbbares Tumorprädispositionssyndrom. Periphere Nervenscheidentumore (PNST) sind ein Kennzeichen von NF1. Plexiforme Neurofibrome (PNF) sind für NF1 charakteristische Neoplasmen, die häufig entstellende Auswirkungen (z.B. im Gesicht) haben und als Präkanzerosen gelten. Frühere Studien haben gezeigt, dass PNF im Gesicht (FPNF) einen Einfluss auf die Form der Gesichtsknochen haben. In dieser Studie werden die Abweichungen der Unterkiefersymmetrie von den kephalometrischen Referenzebenen unter Berücksichtigung der Topografie der FPNF untersucht.

**Material und Methoden:**

Die posterior-anterioren (PA) Kephalogramme von 168 Patienten mit NF1 wurden untersucht. Es wurden drei Gruppen verglichen: Patienten mit FPNF (n=74), mit disseminiert kutanen Neuro-fibromen im Untersuchungsgebiet (DNF, n=94) und eine Kontrollgruppe ohne NF1 (n=23). Die PNF-Gruppe wurde hinsichtlich der Lokalisation und Ausdehnung der fazialen PNST subtypisiert. Es wurden typische kephalometrische Referenzpunkte des Unterkiefers bestimmt (Kondylus, Antegonion und Menton).

**Ergebnisse:**

Die skelettalen Messpunkte des Unterkiefers bei FPNF-Patienten unterscheiden sich oft signifikant von denen der DNF-Gruppe. Ein lokaler Effekt der PNF auf die Relation der Messpunkte zu den Referenzebenen wird durch die Studienergebnisse plausibel gemacht. Unterschiede in den Abständen der bilateralen Messpunkte zu den Ebenen bei FPNF-Patienten können auf ein benachbartes PNF hinweisen. Die Untersuchungen belegen, dass der Tumortyp (FPNF) und die Anzahl der von FPNF betroffenen Äste des Nervus trigeminus mit signifikanten Abweichungen des Unterkiefers von der Symmetrie auf PA-Projektionen korrelieren können.

**Schlussfolgerung:**

Die vorgestellte Studie zeigt, dass bei NF1-Patienten mit FPNF auf standardisierten Röntgenbildern charakteristische Muster der Unterkieferdeformität gemessen werden können. Unterkieferdeformitäten, die auf standardisierten Röntgenbildern abgebildet werden, können erste Hinweise auf eine bisher nicht erkannte NF1 sein. Es wurde nachgewiesen, dass typische Asymmetrien der Messpunkte zu den Referenzebenen Indikatoren eines PNF sind. Unterschiede innerhalb der trigeminalen Tumorausbreitungsmuster werden in den Messwerten angezeigt. Tumor-assoziierte Veränderungen des Unterkiefers sollten in den Klassifikationssystemen pathognomonischer, dia-gnostisch wegweisender Knochenbefunde bei NF1 berücksichtigt werden. Die radiologischen Befunde liefern Anhaltspunkte für die Planung von Unterkieferosteotomien bei NF1-Patienten, insbesondere für die Beurteilung von Gesichtsregionen, die typischerweise durch Tumor-ausbreitung stark vaskularisiert sind. Darüber hinaus sind die radiologischen Befunde ein Hinweis auf einen Tumor, der möglicherweise in die angrenzende Kau- und Mimikmuskulatur eindringt und diese zerstört, was sich auf die chirurgischen Maßnahmen (Funktion, Ästhetik und Wundheilung) auswirken kann.

## Introduction

Neurofibromatosis type 1 (NF1) is an autosomal dominant hereditary tumor predisposition syndrome. NF1 is a monogenic disease caused by a mutation on chromosome 17q11.2 [1]. NF1 patients are predisposed to developing certain neoplasms, particularly peripheral nerve sheath tumors (PNST) [2]. Typical tumors in NF1 patients arise due to loss of function of the *NF1* gene in Schwann cells of peripheral nerves and are termed neurofibroma [3]. Neurofibromas are divided into subtypes according to histological criteria, primarily into cutaneous (synonym: dermal) and plexiform neurofibromas (PNF). PNF can occur ubiquitously in the peripheral nervous system and are considered precancerous. Neurofibromin, the gene product of the *NF1* gene, participates in controlling the pathway of rat sarcoma (RAS) homologue in man [4]. Neurofibromin has tumor suppressor characteristics [5]. Loss of functional neurofibromin causes impaired RAS control and allows increased cell growth [6]. However, *NF1* has further functions beyond RAS pathway control [7]. The changes in the skeleton of NF1 patients (e.g., short stature, early osteoporosis) are non-oncological characteristics of the entity [8]. In the oral and maxillofacial areas, plexiform neurofibromas (PNF) may develop, which may cause severe handicaps. It is known that facial PNF (FPNF) can be associated with changes in adjacent bones [9]. PNF-associated deformities of the maxillofacial bones are highly variable and usually develop unilaterally [10]. They evoked medical interest soon after the disease was defined [11] and prompted repeated attempts at classification [10], [12]. PNF-associated deformities may contribute significantly to patient disfigurement [13], may cause progressive bone remodeling and loss [14], [15], may severely impair functions such as chewing [16], [17], and are difficult to treat [1]. In some cases, jaw deformity and suspected intraosseous tumor spread caused diagnostic problems for differentiating a malignant peripheral nerve sheath tumor [18], [19]. This study analyzes mandibular shape of NF1 patients in a standardized radiological projection to detect patterns of mandibular deformation dependent on facial tumor spread. The study aims to contribute to the facial phenotype of the NF1 patient, with particular emphasis on the impact of facial PNST on mandibular shape [20], [21], [22], [23], [24], [25].

## Material and methods

### Patients and cephalograms

The posterior-anterior (PA) cephalograms of 168 patients with NF1 were examined [males (m): 86 (51.19%); females (f): 82 (48.80%), f/m=1:1.04; age: mean value (MV) 37.9 years (ys), range: 4.17–63.4 ys (females); MV: 37.8 ys, range: 3.81–69.8 ys (males)]. Total number of NF1 patients aged 18 ys or more (“Age over 18 years”) was 119 (70.84%) and <18 ys was 49 (29.16%). The number of *male* NF1 patients ≥18 ys of age was 54 (45.38%, MV: 37.79 ys, SD 13.75, minimum: 19.25 ys, maximum: 69.83 ys) and <18 ys was 32 (65.30%, MV: 10.67 ys, SD 3.81, minimum: 4.17 ys, maximum: 17.58 ys). The number of *female* NF1 patients ≥18 ys of age was 65 (54.62%; MV: 39.97 ys, SD: 11.37, minimum: 18 ys, maximum: 63.42 ys) and <18 ys was 17 (34.70%; MV: 12.32 ys, SD 3.25, minimum: 6.92 ys, maximum: 16.42 ys). 

All NF1 patients met the currently recommended diagnostic criteria [26]. Most patients had been examined for consecutive surgical treatment of facial PNST, so histological findings attested to the tumor [2], [14], [17]. The patient group was divided according to the PNST type (Table 1 (Tab. 1)). PNST were divided into two main groups: PNF and cutaneous. Patients with a facial PNF constituted the PNF group (N=74). In addition to histological findings, estimation of PNST extension was recorded through careful clinical examination and evaluation of magnetic resonance imaging (MRI) of the head and neck region. Only unilateral FPNF were diagnosed in patients of this study. The assignment of patients to individual FPNF subgroups considers the observation that although the facial tumors can be classified according to the dermatomes of the fifth cranial nerve, the extension of the tumors can lead to overlapping of the cutaneous territories and thus combinations of adjacent dermatomes may characterize the facial phenotype more precisely in individual cases. The maximum spread of PNF of one side of the face involves all three trigeminal branches and is called hemifacial PNF. The assumption is that the pattern of FPNF indicates the outgrowth of tumorous Schwann cells (meaning biallelic loss of function of the *NF1* gene in affected nerve sheath cells) which originally arose in close vicinity during the embryonic phase ([27], p. 81-107) and presently define a soft-tissue space-occupying lesion in a terminal area of the cranial nerve [28], [29], [30]. Tumor combinations of only the first and third trigeminal branches did not occur in this study. This observation supports the view on genetically altered, topographically closely associated nerve sheath cells of developmental fields constitutive for a dysplastic/neoplastic facial region in patients with FPNF.

NF1 patients who had *not* developed FPNF were distinguished from this group (N=94). These patients could have optionally developed disseminated cutaneous neurofibromas, including the facial skin, but had no FPNF. The patients with disseminated neurofibroma (DNF) define the DNF group [20]. A previous PA projection cephalometric study of NF1 patients has demonstrated that no significant facial skeletal asymmetries are to be expected in DNF patients [20]. In addition, a cephalometric control group of subjects with ideal occlusion without orthodontic or surgical treatment was analyzed (N=23). This group has been described in detail [21]. Exclusion criterion for the investigation of the NF1 study groups was medical history of surgical skeletal interventions in the craniofacial area, except for dentoalveolar procedures. Furthermore, individuals with known other diseases with a potential impact on skull development were excluded. If a measuring point could not be identified with certainty in an individual case, this measuring point was not considered in the evaluation. 

The X-ray examination of the skull is part of the standardized diagnostic work-up to screen for osseous pathological findings. Most of the X-ray examinations were fixed on film and were digitized for these examinations. The procedure has been described in detail elsewhere, as has the standardized radiological examination and calculations of measurement errors [20], [21], [22], [23]. To capture age effects, measurements were performed of individuals both irrespective of age and restricted to age ≥18 years. For the study, the data were anonymized, and the investigators were blinded to the diagnostic data.

### Measurement

**Figure 1:**
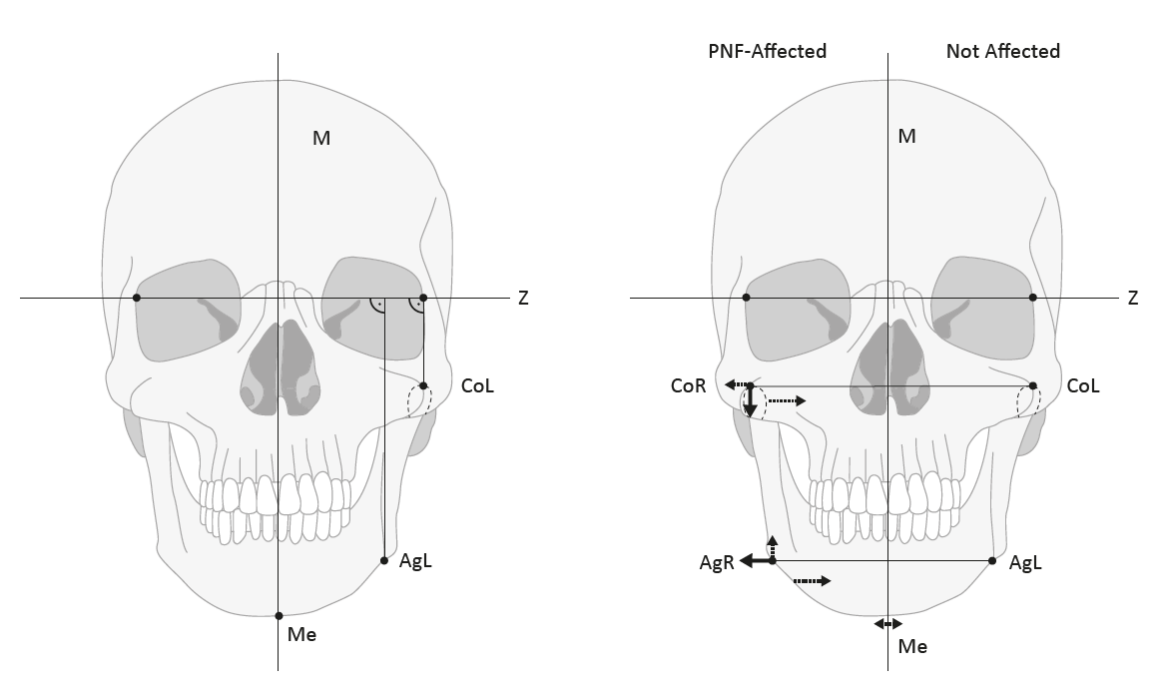
Illustration of radiological cephalometric landmarks (left) and synopsis of measurement results in FPNF group (right). The measurement points are marked on a schematic representation of the skull surface. In this position, the cranial border of the condylar process is not visible with the bare eye and therefore is shown in dashed outline. The individual lines CoR-M, CoL-M, AgR-M and AgL-M are defined by their perpendicular intersection with the median-sagittal axis. The total distance CoR-CoL is defined in transversal diameter and is calculated from a line connecting the highest point of left and right condyle (Intercondylar distance). The line AgR-AgL connects the highest points of the indentation of the mandibular base close to the angle. On the right side, a schematic representation illustrates the position changes of the measurement points in patients with facial plexiform neurofibromas. In patients with FPNF, the condyle is positioned more laterally and caudally on the affected side compared with the unaffected opposite side. Patients who have developed PNF only in the third trigeminal branch deviate from this pattern. The position of the condyle in this group (NV3), which is closer to the M plane, differs significantly from that of other FPNF groups. This finding is illustrated by the medially directed dashed arrow below the measurement point. This deviation is in the same direction as the more medial position of the antegonion in this group (NV3-FPNF, see below). In patients with FPNF, the antegonion measurement point is shifted cranially and laterally. This effect is particularly evident in patients with hemifacial FPNF. The position of the antegonion on the tumor-affected side deviates from this scheme in patients with PNF manifesting only in the third trigeminal branch. In this case, the measurement point of the tumor side deviates towards the median sagittal. The different position of Ag on the affected side in patients with FPNF restricted to the mandibular nerve (NV3-FPNF) is indicated by the dashed arrow below the measuring point AgR (right side is the FPNF side in this illustration). The findings are discussed in more detail in the text.

**Figure 2:**
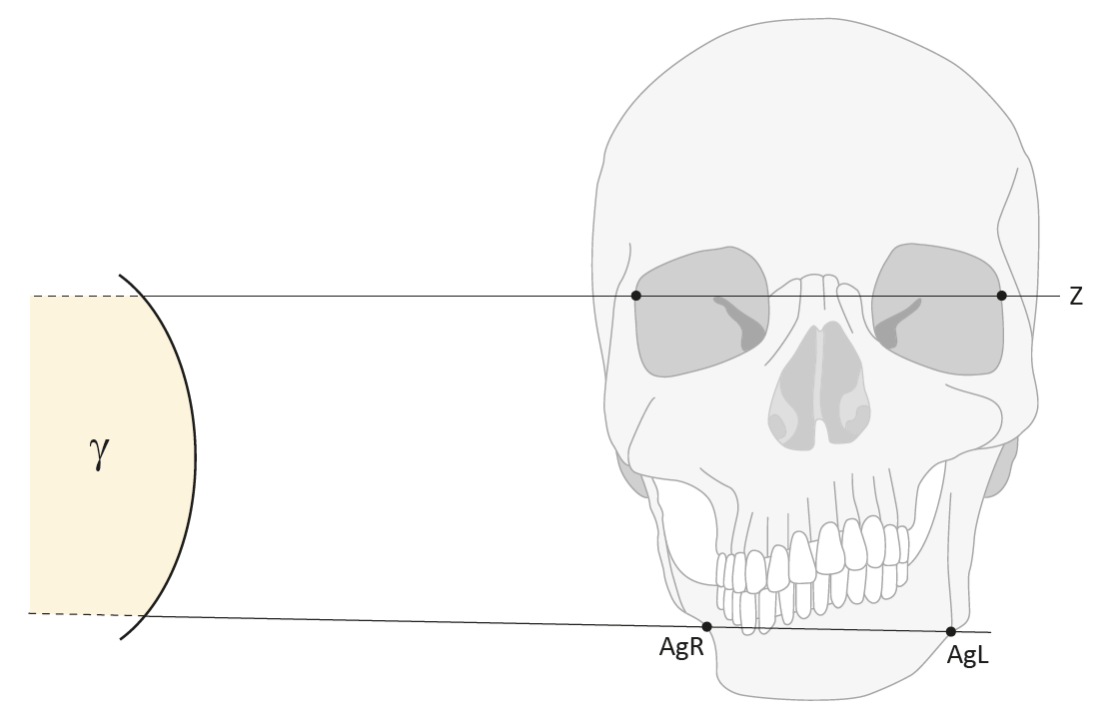
Simplified schematic illustration of the skull viewed from the front. The line labeling “Z” defines a plane parallel to the horizontal. The measuring points AgR and AgL are usually not parallel to the Z plane: the angle of both distances defines gamma. In FPNF patients, Ag of the affected side frequently is more cranially situated than on the non-affected side. For didactic reasons, the drawing reproduces a significant maxillomandibular dysplasia, which causes a higher antegonion notch on the right side with a deformed mandibular ramus. The skeletal as well as the dentoalveolar conditions of the NF1 patients, namely the FPNF patients with tumors of the mandibular trigeminal branch, may deviate from the simplified proportions presented here.

In the cephalograms, a horizontal plane (Z-plane) was defined using lateral reference points of the orbits [20], [22], [23]. The midline of the skull was determined by the perpendicular connection of *crista galli* with this horizontal midsagittal plane or median plane (M-plane). The cephalometric measurement points ‘Condyle’ (Co), ‘Antegonion’ (Ag), and ‘Menton’ (Me) points were used as reference points for mandibular symmetry assessment. Laterality of bilateral measurement points is identified by description of side (right=R, left=L). In the general evaluations of the groups, the sides of the body are first compared with each other (R/L). In further evaluations of the FPNF patients, the measured values are evaluated according to affected/not affected (A/NA) side. Definitions of landmarks are listed in Table 2 (Tab. 2) and illustrated in Figure 1 (Fig. 1) and Figure 2 (Fig. 2).

The following relationships were quantified for the bilateral measurement point ‘Condyle’ (CoR, CoL): Distance CoR and CoL to the reference planes (M, Z), intraindividual difference of the measured distances (CoR-M minus CoL-M; CoR-Z minus CoL-Z) considering the diagnostic groups, and distance between the two measuring points CoR-CoL (intercondylar distance). 

The following relationships were quantified for the bilateral measuring point ‘Antegonion’ (AgR, AgL): Distance AgR and AgL to the reference planes (M, Z), intra-individual difference of the measured distances (AgR-M minus AgL-M; AgR-Z minus AgL-Z) considering the diagnostic groups, distance between the two measuring points AgR-AgL (width of the mandible), and angle of the crossing lines AgR-AgL and Z-plane. 

Furthermore, the distance of the lowest point of the bony chin, Me from the M-plane was measured. The measurement point is a single value.

In all FPNF patients, laterality of the neoplasm was identified. The dependency of the measured values on the side of the tumor was determined (affected (=A) vs. non-affected (=NA) side). In addition, it was examined whether there is a connection between the respective distribution pattern of the facial tumor and mandibular symmetry in relation to the reference planes. The subdivision of the patient group according to the spread of the tumor in the cutaneous areas of the trigeminal nerve has proven to be a helpful orientation for clinical diagnostics of facial skeletal lesions in earlier studies [21], [22] and was also used here.

### Ethics

All procedures performed in this study involving human participants were in accordance with the ethical standards of the institutional and/or national research committee and with the 1964 Declaration of Helsinki and its later amendments or comparable ethical standards. Data were anonymized prior to analysis, and the investigators studying the radiographs were blinded for diagnosis and the identity of individuals. The investigations of anonymized data were performed in accordance with Hamburgisches Gesundheitsdienstgesetz (Hamburg Health Services Act). This type of investigation does not require the approval of the local ethics committee. The examinations are part of a scientific thesis to meet the requirements for obtaining the degree of Doctor of Dentistry (GC).

### Statistics

Arithmetic mean (mean value(s), MV), standard deviation, and paired and unpaired t-tests were calculated. Significance level was set at *p*<0.05 (p<0.05=*, p<0.01=**, p<0.001=***). Calculations were carried out with SPSS (IBM, Armonk, USA).

### Error analysis of the measurements

The calculation of the method error according to Houston was used to prove the reproducibility of the determination of the measuring points. A reliability coefficient >0.9 indicates a high degree of reproducibility of the identification of measurement points and the measurement results. This value was obtained for various cephalometric measurement points in this study and has been described in detail elsewhere [20], [21], [22].

### Presentation of results

In the tables, the measurement values of the control group [21] and the DNF group [20] are presented first. Furthermore, in the FPNF group, subgroups were defined according to FPNF spread and the measured values of these groups were compared with each other. The measurement results of the PNF group (total group and specified by subgroup) are presented in relation to these comparison groups (Tables 3–14).

## Results

### Condyle

#### Distances of condyles to Z-plane (Co-Z)

The vertical distances of the condyles (CoR-Z, CoL-Z, CoA-Z, CoNA-Z) to the horizontal plane (Z-plane) do not differ significantly in R/L comparisons in all three total groups. The small lateral differences (R/L) in FPNF patients (total group) suggest the vertical position of the condyles to the reference plane (distance Co-Z), balance out in the aggregate, probably due to the equal distribution of tumors on both halves of the body. However, several differences in the group comparisons are significant, if the total group of FPNF patients is differentiated according to tumor-affected and non-affected side, as well as according to the topography of the tumor (FPNF subgroups). The distance Co-Z is longer on the affected side, meaning the condyle’s position is more caudally on the affected side. If the patients with FPNF are further subdivided according to a facial distribution pattern of the tumor defined by the trigeminal dermatomes, this difference remains equally significant in most subgroups. The numerical largest intraindividual difference A/NA (MV) is measured when comparing patients’ condyles with hemifacial PNF. However, PNF localized exclusively in the midface (2^nd^ trigeminal branch) has no significant influence on vertical condylar position. It is interesting to note that PNF localized exclusively to the third branch has no influence on this distance (Table 3 (Tab. 3)). 

#### Comparison of intraindividual differences of vertical distances (CoR-Z minus CoL-Z; FPNF: CoA-Z and CoNA-Z)

In the next analysis, the differences of the bilaterally registered distances Co-Z were calculated intraindividually. In DNF patients, the sign of the difference (+/–) was defined by ranking of body side (Right minus Left). In FPNF patients, the distances were related to side of tumor (A minus NA).

Then, the differences between the diagnosis groups were compared. In NF1 patients aged ≥18 years, the differences between the diagnostic subgroups hardly had any statistically significant effects. Statistically significant effects were registered when comparing FPNF patients (CoA minus CoNA) with manifestations in the 1^st^ and/or 2^n^^d^ trigeminal branch and those with hemifacial PNF. This result was confirmed when NF1 patients were included in the evaluation without age restriction. 

Patients without age restriction were recruited for further calculation. In this study, the highly significant difference in the measurement results of these two PNF groups compared to the hemifacial PNF group was confirmed (Table 4 (Tab. 4)). In all cases, the most cranial point of the condyle of the affected side of the hemifacial PNF group is farther away from the horizontal reference plane than in the PNF comparison groups (positive measurement values). Age effects must be considered when evaluating these measurements. Only the group of patients exclusively affected in the third branch is excluded from the enlarged CoA-CoNA difference on the tumor side. The difference is highly significant (*p*=0.002).

#### Total distances of condyles (CoR-CoL, intercondylar distance)

The total distance CoR-CoL was 105.84 mm (SD 5.76) in the control group (n=21), 103.32 mm (SD 6.76) in the DNF group (n=94), and 100.86 mm (SD 8.05) in the FPNF group (n=72). The differences in intercondylar distances comparing the ‘control’ vs. ‘DNF’ groups are statistically not significant (*p*=0.077). The intercondylar distances of the FPNF group and the control group are significantly different (*p*<0.002), as are the distances of the FPNF group and the DNF group (*p*=0.039). The intercondylar distances of the FPNF group are the shortest. This results in a skeletally narrower face of these patients in the region below the zygomatic arches. Further analysis focused on the identification of the effect of the respective condylar side to the total distance, especially considering the affected side in patients with FPNF. To prepare values for comparisons, the distances CoR-M minus CoL-M were calculated individually (see above). 

### Distances of condyles to M-plane (Co-M)

The differences of the transversal distances of the measurement point “condyle” to the median sagittal are without statistical significance in the total group NF1, the control group, and in the DNF group. The FPNF subgroups differentiated into facial tumor locations also show no statistically significant difference in this dimension when differentiated according to the criterion of affected vs. nonaffected side. Only the patients with hemifacial PNF have a statistically significantly greater distance of the condyle of the tumor side from the reference plane than the non-affected condyle (Table 5 (Tab. 5)). In the following analysis, it was examined whether the individual differences of the measured distances (distance Right side minus Left side and affected side minus non-affected side, respectively) have group-specific differences.

### Comparison of intraindividual differences of horizontal distances (CoR-M minus CoL-M)

This calculation shows no statistically significant differences in all groups when individuals over 18 years of age are included. Thereby, the comparison of the MVs of some groups indicates considerable differences of the measured distance. Apparently, the range of variation of the condylar position to the median sagittal is so large that the differences balance out across all groups of adults. However, some differences within the FPNF subgroups become apparent when individuals of all ages are included. Statistically significant differences are registered when comparing several subgroups with the group of hemifacial tumor-affected individuals. In contrast to the group of hemifacial PNF patients with the condyle on the affected side farther away from the median sagittal plane, in two of the three subgroups the distances Co-M are negative, i.e., the condyle adjacent to the tumor is positioned closer to the reference plane than the condyle on the non-affected side. In detail, both patient groups with PNF in the 1^st^ and/or 2^nd^ trigeminal branch (n=39), and those with manifestations in the 2^nd^ and/or 3^rd^ branch or all three branches (n=33), have a positive difference value of the horizontal distance Co-M, meaning that the mandibular condyle of tumor side is farther away from the midline than the condyle of the non-affected side. However, the differences between both groups are not significant (*p*=0.665). Further comparison of the FPNF groups shows differences in condylar position. The group of patients affected in the 2^nd^ and/or 3^rd^ branch (n=17) has a condyle positioned *closer* to the midline on the tumor side. In contrast, the condyle of patients with hemifacial PNF (n=16) is farther away from the midline than that of the opposite side. The positional differences are highly significant (*p*=0.009). 

The farther lateral position of the tumor-sided condyle of patients with hemifacial PNF (5.907 mm) is significantly larger than the tumor-sided condyle position of the patient group with PNF manifestation limited to the 1^st^ and/or 2^nd^ trigeminal branch (1.042 mm, *p*=0.013) (in both groups, the condyle of affected side is farther away from midline compared to non-affected side). The differences in condyle position of tumor side are particularly striking when patients with PNF restricted to the 3^rd^ trigeminal branch (n=9) are compared with patients who developed hemifacial PNF (n=16). While the former group has a condyle positioned closer to the median sagittal plane on the tumor side in the intraindividual side comparison (–4.813 mm), NF1 patients with hemifacial PNF have a condyle significantly farther away from this reference plane on the tumor side (5.907 mm, *p*=0.007). Table 6 (Tab. 6) summarizes the results.

### Antegonion

#### Distances of antegonion to Z-plane (Ag-Z)

The vertical distance Ag-Z shows no differences in the comparison of the total groups if only individuals older than 18 years are included. In the intraindividual side comparison, a quantitatively small, statistically significant difference is demonstrated in the total group of NF1 patients: The distance is greater on the side where the tumor in PNF patients is located. This difference is confirmed in the side comparison of the FPNF subgroups. With the additional inclusion of individuals <18 years of age in the intraindividual comparisons, the statistically significantly longer distance of the affected side from Z-plane is confirmed. When analyzing the subgroups, this finding is an effect of the FPNF group (*p*=0.001). Within the FPNF group (all ages), the distance Ag-Z on the affected side is equal in size or greater. The findings reach statistically significantly different values only in the two groups with PNF of the 1^st^ and/or 2^nd^, and 2^nd^ and/or 3^rd^, trigeminal branch (Table 7 (Tab. 7)).

#### Comparison of intraindividual differences of vertical distances of reference points ‘Antegonion’ to Z-plane (AgR-Z minus AgL-Z, AgA-Z minus AgNA-Z, respectively)

In the next step, the differences of the distances Ag-Z plane (AgR-Z minus AgL-Z) were calculated intraindividually. In the entire group of individuals >18 years, the side difference in favor of the right side is only 0.572 mm. From this result, it can be concluded that the lateral differences of the vertical distances of the jaw angle measuring point to the horizontal reference plane are quantitatively small in the overall group. Significant lateral differences in the diagnostic groups may indicate a local effect of the PNST. The high minimum and maximum values of the total group refer to findings recorded in the FPNF group. The group comparisons of the distance differences showed no significant differences between the diagnostic groups (Table 8 (Tab. 8)).

#### Total distances of antegonion (AgR-AgL)

The transverse dimension of the mandibular angle region in over 18-year-olds, measured as the distance AgR-AgL, is greatest in the control group, followed in descending order by the DNF and the NF1 total group. The differences in MV are significant in the individual comparison of the diagnostic groups. The MV of the AgR-AgL distances of the FPNF group is lower than the values of the previous three groups, i.e., the transverse mandibular dimension is significantly lower in the mandibular angle region of the FPNF patients than in the control group and the DNF group. Further studies on the influence of tumor localization on the measured values of the FPNF group show that patients with hemifacial tumors differ significantly from the comparison groups in the measured distance (Table 9 (Tab. 9)).

#### Distances of antegonion to M-plane (Ag-M)

The mean values of the distances Ag-M of the NF1 total group (age ≥18 years) differ significantly from those of the control. However, concerning laterality of findings, this difference reveals only on the right side. The difference is confirmed when comparing the DNF group with the control group. It follows that the measurement point Ag of the NF1 patients is closer to the median sagittal plane, in other words, the entire group has a narrower jaw in this region. The MVs of the horizontal distances of the measurement point Ag from the midline in the FPNF group are below those of the DNF group for both the affected and the non-affected side. However, the differences are only significant for the non-affected side compared to both the DNF and control groups.

In the total group of FPNF patients, the antegonion measurement point on the tumor side is farther away from the median sagittal than on the non-affected side. The distance AgA-M and AgNA-M is significantly shorter for the total FPNF group than in the control group (*p*=0.000 and *p*=0.001). In contrast, the MVs of the total FPNF group compared with the DNF group are different only for the non-affected side, which is also shorter than in the DNF group. The lack of difference in the comparison of the FPNF and DNF groups regarding the affected side of the jaw suggests that the differences balance out overall. However, further differentiation of the FPNF group could disclose differences in tumor effects on the position of the measurement point that remain hidden in the overall analysis.

Compared to the control group, FPNF subgroups reveal a statistically significant shorter distance Ag-M for three of five groups concerning both affected and non-affected side.

Significant differences of the measured distances are demonstrated in the hemifacial PNF group and those with exclusive affection of the 3^rd^ branch, both compared to the control group and DNF group. However, these differences affect only the affected side in one group (3^rd^ branch affected: Ag-M shortened) and only the non-affected side in the other (hemifacial PNF group: Ag-M shortened) (Table 10 (Tab. 10)). The opposing alignment of the measuring point displacement Ag relative to M between the groups “3^rd^ branch PNF” vs. “all 3 branches PNF” deserves further analysis. 

For the intraindividual symmetry comparison of the Ag-M distances, *all* individuals were evaluated. The comparison of the distances Ag to M-plane *within* the main diagnostic groups only shows a quantitatively small, significant left-right asymmetry in favor of the left side in the DNF group. This effect has already been reported and discussed [21]. In a second step, all FPNF patients were differentiated according to affected side vs. non-affected side. In the FPNF groups, the distances Ag to M of the affected side were statistically significantly increased only in the groups “2^nd^ and/or 3^rd^ trigeminal branch affected” and those with hemifacial PNF. The shortened distance from the affected side to M is striking in the FPNF patients who were only affected in the 3^rd^ branch. However, the difference is not significant (Table 11 (Tab. 11)).

#### Comparison of individual differences of horizontal distances to M-plane (AgR-M minus AgL-M)

This calculation examined whether the MVs of the group-specific intraindividual differences in the Ag-M distances have statistically significant different values.

The comparison of the entire groups showed no differences, both when excluding <18 years of age and when analyzing the entire group. Significant differences became apparent in the FPNF subgroups >18 years of age, which are attributed to the greater distance from Ag to M on the tumor-affected side in patients with mandibular branch PNF. Patients with hemifacial PNF show the largest deviations of the measured distances compared to control, DNF, and FPNF subgroups.

The striking differences in the transverse relation of Ag to M in patients with FPNF affecting only the 3^rd^ branch of the trigeminal nerve (n=9) and those with hemifacial PNF (n=17) were further specified. In FPNF patients with tumor growth restricted to ‘NV3’, the mean value of the differences ‘Ag-M affected side’ minus ‘Ag-M non-affected side’ is –5.25 mm (SD 6.43, SEM: 2.14), which means that the tumor side is closer to the M plane and appears flattened. In patients with hemifacial PNF, the mean value of the lateral differences between the affected and non-affected side of the Ag-M segment is 6.51 mm (SD: 6.66, SEM: 1.61), i.e., the bone is significantly wider in the jaw angle area of the affected half of the face and thus farther away from the midsagittal than on the non-affected side. Patients with hemifacial PNF have a more ‘bulged’ mandible in the jaw angle area. The differences between these two diagnostic groups are highly significant (*p*<0.0001(***)) and indicate an effect of the respective tumor spread on the transverse position of the mandible. Tumors exclusively manifesting in the 3^rd^ branch of the trigeminal nerve limit the lateral extension of the mandible on the affected side. In contrast, hemifacial PNF have the characteristic of being associated with a significantly more laterally located Ag, presumably an effect of mass, muscle destruction and weight (Table 12 (Tab. 12)).

#### Angle of lines AgR-AgL and Z-Plane

In a comparison of the total groups (inclusion criterion: >18 years old) there are statistically significant differences in the angle between FPNF total group and control (*p*=0.019) and DNF group (*p*=0.002). The angle is larger in FPNF patients than in control and DNF patients. On the other hand, there are no noticeable differences in this angle when comparing the DNF vs. control groups. The comparisons within the FPNF subgroups show no statistically significant differences in the angle size AgR-AgL/Z-plane. The inclusion of individuals <18 years of age does not alter results of group comparisons (Table 13 (Tab. 13), Figure 2 (Fig. 2)).

### Menton

#### Size of the deviations from Menton (Me) relative to the M-plane

For the control group (N=23), the MV is 1.96 mm (minimum: 0.25 mm, maximum: 6.31 mm, SD: 1.88). The values of this group change only insignificantly when only >18 years are considered (MV: 2.07, minimum: 0.25 mm, maximum: 6.31 mm, SD: 1.93, N=21).

In DNF patients (N=91), the MV of menton deviation is 2.77 mm (minimum: 0 mm, maximum: 8.94 mm, SD=2.11). If only >18 years are considered in this group (N=73), then the MV is 2.95 mm (minimum: 0 mm, maximum: 8.94 mm, SD=2.24).

In FPNF patients (N=75), the MV of menton deviation is 2.94 mm (minimum: –14.24, maximum: 21.29 mm, SD=6.23). If only >18-year-olds are considered in this group (N=44), then the MV of Menton deviation is 2.44 mm (minimum: –11.95, maximum: 21.29 mm, SD=6.38).

If only the 3^rd^ trigeminal branch is tumorous altered (N=9), the MV of menton deviation is 0.26 mm (minimum: 11.03 mm, maximum: 16.43 mm, SD=8.66). If only >18 years are considered in this group (N=6), then the MV is –4.11 mm (minimum: –11.03 mm, maximum: 4.28 mm, SD=6.18).

If all three trigeminal branches of one side are affected (N=18), the MV is 5.74 mm (minimum: –4.83 mm, maximum: 21.29 mm, SD=7.95). These values hardly change if only over 18-year-olds (N=11) are considered (MV: 5.92 mm, minimum: –483 mm, maximum: 21.29 mm, SD=7.98). 

#### Group comparisons

In the group of >18 years (N=43) the distance from the lowest point of the bony chin to the median sagittal plane deviates in statistically significant values both in the DNF and the control group. The distance Me-M is larger in the FPNF group than in the DNF and control groups.

However, the comparisons of the FPNF groups differentiated according to the regional characteristics of the facial tumor do not result in any statistically significant differences for those >18 years of age. If the <18-year-olds are included in the comparisons (“all ages”), the results of the overall group comparisons are non-affected by this criterion. 

Also in this study, individuals with an age below eighteen years were included in a further step of the analysis. In this numerically expanded group, statistically significant differences in Me deviation were detected in the comparisons between FPNF groups and hemifacial PNF. When comparing the hemifacial PNF group with the subgroups with predominant localization of tumors in the midface, the deviation of menton from the median sagittal was significantly greater (*p*=0.012; *p*=0.009), irrespective of deviation direction (Table 14 (Tab. 14), Figure 1 (Fig. 1)). It is interesting to answer the question whether in FPNF patients a direction of menton deviation can be determined depending on the tumor side.

#### Deviations of the measuring point Menton to the affected/non-affected side in FPNF patients

In the FPNF total group (N=73), the measurement point deviated to the affected side in 60.3% of the sample, and to the non-affected side in 38.4%. The subdivision of the FPNF group according to affected trigeminal branches showed clear differences in the frequencies to which side the deviation had occurred. If the 1^st^ and/or 2^nd^ branch was affected (N=22), the measurement point was on the affected side in 59.1% (non-affected: 40.9%) of cases. If the 2^nd^ and/or 3^rd^ branch was affected (N=9), the shift was always on the FPNF side (affected side: 100%). If the patients were hemifacial affected by the FPNF (N=18), then menton was located on the affected side in 77.8% of the cases (non-affected side: 55.6%). In contrast to this distribution of menton deviations, in patients affected by FPNF in the 3^rd^ trigeminal branch only (N=9), the measurement point was shifted to the tumor side in 44.4% of the cases (non-affected side: 55.6%).

The directions of the deviations of menton from the M-plane, analyzed in groups, were compared with each other. The group comparison between the group “1^s^^t^ and/or 2^nd^ branch” (FPNF of the midface, N=22) vs. group “2^nd^ and/or 3^rd^ branch” (mid/lower face, N=9) is statistically significant (*p*=0.022*): The more caudal the tumor extends, the more frequently menton is located on the tumor side. On the other hand, the distribution differences of the measurement point dependent on the tumor side are not significant (*p*=0.63 and *p*=0.490) in both group comparisons (“1^st^ and/or 2^nd^ branch” (N=22) and “2^nd^ and/or 3^rd^ branch” (N=9)) against the hemifacial PNF group (N=18). It follows that in FPNF patients with extensive tumor manifestations and multiple affected trigeminal branches, menton is located on the tumor side in most cases. A shortened mandible on the tumor side is often observed in these cases. In contrast, the group comparison of the FPNF patients affected only in the 3^r^^d^ branch with the group of hemifacial FPNF patients, proves the statistically significant difference of the chin tip displacement (*p*=0.036*): Menton deviates to the non-affected side in more than half of the patients FPNF-affected only in the 3^rd^ branch.

## Discussion

Numerous studies described individual, detailed findings concerning the frequently very conspicuous bone deformations of the facial skull in NF1. Other authors analyzed the coincidence of certain soft tissue and osseous changes, and some of the findings were assessed as pathognomonic in NF1 [9], [10], [11], [14]. More recent radiological examinations identified combinations of skeletal changes in the jaw and joint regions as being an indicator of a disease-typical tumor of NF1, the PNF [9]. The quantitative examination results presented here provide guidance for the radiological evaluation of deviations in mandibular symmetry in NF1 patients as an indicator of this neurogenic tumor in topographic relation to the bone. Consideration of the spread of FPNF, defined as affection of the facial soft tissues by the branches of the trigeminal nerve (diagnostic groups), as a factor influencing mandibular symmetry, allowed the conclusion that the extent of bone change is highly dependent on whether the 3^rd^ trigeminal branch is tumorous. Tumors near the base of the skull have the greatest effect on bone changes. The present investigation reveals a pattern of mandibular deformation can be measured in NF1 patients with FPNF despite considerable phenotypic variability. The prerequisite is the use of a standardized radiological PA projection of the skull and meticulous cephalometric analysis, combined with a precise classification of facial tumor extension. On the other hand, it has been shown that NF1 patients without FPNF do not show significant deviations in mandibular symmetry [21]. The results could have significance for the anthropological assessment of NF1 patients (for example in the discussion about the so-called ‘NF-face’ [31]), the identification of regions suspected for being invaded by a tumor defined as a precancerous lesion [9], and the planning of orthognathic surgical interventions [20], [21]. As a general result, a facial PNF can regularly be detected in a topographical relationship ipsilateral to the deformed mandibular side, which preferentially affects the 3^rd^ branch of the trigeminal nerve.

### Condyle

The intercondylar distance of DNF patients showed no significant differences from the control group. The MV of the distances of the present study in control group and DNF group corresponded well to published anthropological/radiological data [32]. The vertical growth patterns of the skull have no influence on the intercondylar distance [33], [34]. The finding that the slight tendency of DNF patients to develop a “long face” [20] is without any influence on the intercondylar distance and fits in with these results. In contrast, the transverse dimension of the skull in FPNF patients is defined by a comparatively shorter intercondylar distance. The skeletal narrowing of the face in this region correlates with measurement results for the “zygoma” measurement point in this patient group [23].

It was also shown that in FPNF patients the condyle is positioned medially, primarily on the tumor-affected side (CoA-M). In contrast, when FPNF is developed, the total vertical distance (CoA-Z) on the affected side is longer. Radiological analysis of the skeleton under standardized conditions showed a significant difference between controls and FPNF patients. However, it is not possible to determine from radiographic examinations whether this is a primary (independent) effect of bone formation in a tumorous environment, secondary effects of the skeleton (e.g., excess secretion of growth factors [2]) due to contact with neurofibroma, or a combination of several conditions. Furthermore, impaired muscular activity due to tumor-invaded, often destroyed masticatory muscles attaching to the *processus condylaris* and other mandibular regions could also contribute to the shortening of the distance Co-M and increasing the distance Co-Z on the tumor side of FPNF patients [15].

Considerable objections concerning the position of the articular processes of the mandible were raised when assessing skeletal changes of the facial skull of NF1 patients on plain radiographs [25]. It was conceded that qualitative changes of the jaws, for example the PNF-dependent deformation of the articular process [9], could merely be described by means of orthopantomogram [25]. However, this examination technique is not considered suitable for adequately measuring subtle changes such as the sagittal position and diameter of the condyle. For this purpose, three-dimensional imaging of the bone was required in NF1 skull research [25]. On the other hand, the two-dimensional cephalometric analysis allows the symmetry comparison of the joint position in a standardized radiographic PA orientation of the central beam, and at low radiation exposure of patients. Detailed three-dimensional studies provided evidence of a significantly larger condyle that is developed, measured in the PA direction in NF1 patients without FPNF [25]. 

It has been suggested that this effect is a constitutive skeletal feature of the NF1 patient. It remains unknown whether the calculated subtle differences in the position of the articular head in the condylar fossa, and the dimensions of the condyle, have an impact on the chewing function and the aesthetics of the patients. On the other hand, a tumor that is characteristic of NF1, the PNF, has a measurable effect on the vertical and transverse positions of the condyle when the neoplasm develops in the facial regions and adjacent skull, including the temporomandibular region. Furthermore, the current findings support the conclusion that in DNF patients, the position of the condyle relative to the median sagittal plane is equal in terms of symmetry compared to healthy individuals.

### Antegonion

The study reveals changes in the shape of the mandible on PA cephalograms of certain patients with NF1. In these cases, considerable deviations of the measurement point ‘antegonion’ from the median sagittal plane were recorded. Typically, there is a distinct deviation of the Ag-M distance on one of the two sides in FPNF patients compared to symmetrical readings in control and DNF groups. The somewhat farther distance is to be assigned to the tumor side. This statement does not apply to patients who are affected by PNF in the 3^rd^ branch of the trigeminal nerve. The cause of this finding probably is the extent of the tumor, which in mandibular-branch-only affected PNF patients often had extended farther down into the neck region. It can be assumed that tumor expansion and functional soft tissue changes contributed to the altered distance CoA-M in these cases.

### Menton

Under ideal-symmetric conditions, the value of the measuring point ‘Menton’ in the PA cephalometric analysis is 0. Menton measurement point indicates changes of the anterior mandible’s position relative to the median sagittal plane. Measurement values of menton are farther away from the reference plane in NF1 patients, and the interpretation of the results should be done with caution. When analyzing the lower jaw, it is assumed that the outline of the bone is developed largely symmetrical [35] and oriented parallel to the median-sagittal plane. However, in the radiological examination, incorrect positioning of the lower jaw is a well-known problem that can lead to incorrect measurement results. The positioning problem also exists with a three-dimensional representation of the region of interest [20], [21]. According to cephalometric analysis (e.g. on PA radiographs), deviations of the measured values of bilateral measurement points in individual side comparison increase in the cranio-caudal direction. The deviations are understood as physiological adaptations of the individual musculoskeletal system. A “physiologically” wider distribution of the measuring point ‘Menton’ in relation to the median sagittal must also be considered, the cause of which, in addition to adaptive skeletal changes [35], can be an unequal positioning of the articular process in the glenoid fossa [25]. Dentoalveolar changes are another factor that influences measurement results for symmetry assessment. Asymmetries of the mandible in the selected radiological projection can therefore have technical (positioning) but also biological causes, e.g., skeletal pathologies and/or associated soft tissue anomalies. Endocrinologic disorders should be considered when examining skeletal changes [36]. In patients with facial PNF, the clinical interpretation of radiological findings have to consider that the patient’s skeletal deformity (predominantly hypoplasia and narrowing of bones to the median) can be covered by hyperplasia of the tumor-infiltrated soft tissue. In other words, from the measurement results, it cannot be concluded that patients with, e.g., a shortened mandibular body and deviation of the mandible to the affected side, inevitably have underdevelopment of the soft tissues of the ipsilateral (lower) face. Rather, the tumorous soft-tissue masses can compensate the skeletal malformation and even cause tumor-side facial hyperplasia despite hypoplasia of the jaws [37]. On the other hand, local trophic effects are possible in individual cases of producing true hypertrophies of bones or bone sections, including the mandible [38]. However, this finding is rare. In many cases, only a closer clinical examination, especially oral inspection, offers clues to the bony situation. In summary, the test results provide objectified metric data at the menton measuring point for the known assessment that the lower jaw in NF1 patients with FPNF is shifted towards the tumor side in many cases. This result is relevant for planning orthognathic interventions for these patients.

### Symmetry of the facial skeleton

In the assessment of the skull in the view *en face*, symmetry of the face is assumed [21]. Conspicuous deviations from symmetry are consistently registered as individual characteristics. The transition is gradual from small asymmetries that can hardly be considered by physical inspection or instrumental measurements to obviously severe distortions. However, exact skull symmetry does not exist below human visual resolution. Threshold values are therefore considered in anthropometry to define symmetry [21]. Therefore, symmetry of an examination object such as the skull is defined within biological range and asymmetry is determined if the limit values are exceeded.

The measuring threshold is necessarily arbitrary and usually refers to a measurement accuracy claimed to be sufficient for general (clinical) requirements. The discussion of the measuring accuracy of the tool (e.g., in cephalometries irrespective of the device) is an unavoidable part of the error analysis of any radiological procedure aimed to identify (a)symmetry in human beings [21]. 

### Age effects on cephalometric readings

Cephalometric analysis of craniofacial symmetry must consider age effects. For example, mixed dentition of children and adolescents is thought to influence the position of measurement points [32]. Indeed, only few statistically significant changes in condylar position are observed in patients >18 years of age (total group) when comparing bilateral values. However, the differences in intraindividual condylar positions described in the results are preserved in NF1 patients over 18 years of age with FPNF in the same orientation as is calculated for the whole group, including those <18 years of age. The reduced group size, when applying the age limit ≥18 years, is probably one reason for limited statistical validation of the conclusions.

Indeed, while PNF is a common finding in NF1, frequency of facial PNF is not well documented. NF1 patients with FPNF are not usually registered as a distinct entity but rated as part of head and neck PNF, a finding noted in 1.2% [39] to about 30% [40] of NF1 patients, depending on definitions of the study population and criteria of evaluation. However, it was pointed out that mandibular development occurs symmetrically in a healthy organism, because the neural development preceding bone development follows the criteria of bilaterally symmetrical growth and differentiation [35]. Minor mandibular asymmetries in healthy individuals are rated as resulting from skeletal adaptation to functional stress [35]. The presented study shows that mandibular asymmetries are associated with the spread of FPNF.

Early impact of a tumorous trigeminal branch on bone development is very likely because the same pattern of mandibular findings can already be detected in children [9], [14]. The partial alignment of the condyle measurements regarding the distance to the median plane could be an expression of functional adaptation in adults on both body sides. However, the morphological changes in the condyles of NF1 patients with FPNF are often bizarre. The measuring points only allow standardized, point-by-point comparisons and do not represent the outline of the deformed jaw side. Furthermore, the extent of the soft-tissue changes (volume, functional limitations) is not captured by the radiological examination. A review of the study results with a larger patient group and alternative examination techniques, for example CBCT, is desirable. Three-dimensional analysis of the condyle of NF1 patients without FPNF can help identify even small skeletal changes below visibility threshold and assert them as a component of the skeletal phenotype [25]. Asymmetries of the mandible within physiological range of healthy individuals on CBCT are calculated to 1 mm lateral differences by side [32], a finding in the range of results obtained in healthy volunteers and DNF patients on PA cephalograms [20], [21].

### Facial skeletal and soft tissue findings in NF1

NF1 is a chronic disease with often significant growth spurts [1]. Facial PNF usually grow invasively and slowly destroy the adjacent parenchyma. In advanced stages, the facial muscles on the affected side are regularly destroyed and soft tumor masses dominate the motorically restricted (often paralytic) half of the face, which prolapse caudally because of gravity. As a rule, it is clinically impossible to distinguish what part of the bone deformation is primarily due to the congenital differentiation disorder, the periosteal (rarely intraosseous) tumor infiltration, or the tensile forces of the motionless slack hanging tumor mass on the shape of the facial skull (e.g., the lower jaw). Observations in children with facial PNF suggest that characteristic skeletal changes are present soon after birth, such as deepened semilunar incisure, shortening of the mandibular corpus combined with a notching of the basal contour of the mandible anterior to the external angle of the jaw, and retention of teeth [14]. On the other hand, in some cases, the bone change progresses and can lead to destruction of a bone or part of it [15].

Although facial findings in NF1 patients are used as anthropological indicators of certain genetic changes in the NF1 gene [24], Riccardi’s assessment still applies in most cases arguing that NF1 patients usually resemble their parents and do not develop a pathognomonic face ([41], p. 110). The discussion about anthropological characteristics of the face of the NF1 patient focuses on the assessment of predominantly soft-tissue proportions [37], except for the controversially discussed frequency of hypertelorism in NF1 [31] and its possible indication of genetically defined subtypes [42]. However, discussions of NF1-associated hypertelorism are often based on estimates of soft tissue measurements [42].

Radiological studies of the facial skeleton indicate that the diagnosis of NF1 in patients without facial PNF meets requirements to diagnose a face constituted by symmetrically developed bones. However, one must consider the biological framework of bone development, which means minor deviations from strictly mathematically defined geometric criteria may be recorded but usually are below the threshold of visual perception [21]. The findings show any deviations from cephalometric norms [21] that would allow us to address a so-called NF1 face [20], [31]. On the other hand, it is shown here in a cephalometric study, that a tumor characteristic of the disease has an identifiable impact on the facial skeleton in the case of facial manifestations. The impact of FPNF on bone is identified with a simple, standardized radiological examination technique. The tumor may cause typical changes in the adjacent facial skeleton, as well as be associated with changes in reference points to reference planes on the non-affected side, the latter possibly resulting from compensatory positional changes in bone. The skeletal changes are so characteristic that they can be quantified in a simple analysis. The effect of PNF on adjacent bones has been known for a long time [1] and has significance in establishing the clinical diagnosis of the disease [26]. Concerning the orbit, sphenoid dysplasia was recently reclassified as a relevant clinical diagnosis of NF1, but not independent from evidence of ipsilateral orbital PNF. Analysis of the skull below the skull base of the NF1 patient for PNF-associated bony changes has presented numerous details [9], [10]. However, facial bone findings currently are not included in the diagnostic lists of the NF1, presumably because the topographically associated facial PNF are in the foreground of the diagnosis (and severity assessment of the disease). However, in some cases, bony changes in the facial skeleton may precede the diagnosis of NF1 [43]. The presented study proves the statement that a combination of mandibular cephalometric findings can be attributed to the canon of PNF-associated skeletal characteristics of NF1 [44] and may pave the way to establish diagnosis of the disease in individual cases, provided a careful radiological analysis is carried out [43].

PNF often is an invasive and destructively growing tumor [15]. In individual cases, the bone deformation may deviate considerably from the pattern presented here, which is evident in the view of the skull on PA radiographs. In cases with major deviations from skull symmetry, the diagnosis of an osseous lesion, and the examination of whether a malignant transformation of a PNST is arising in a suspected orofacial region associated with bone lesion, are often at the forefront of the diagnosis [18], [45].

Other studies have interpreted local bone changes in NF1 in the concept of syndromal ‘osteopathy’ [46]. The results presented here add to this concept characteristic mandibular changes that can often be expected because of FPNF. In fact, NF1 is also a disease of the skeletal system [8], [37]. However, striking changes in the symmetry of the facial skull appear to be associated with the extent of an FPNF [9], [22], [23]. These changes can be observed as early as infancy [9]. It is likely that the mandibular deformities in FPNF are a consequence of tumor formation already during the embryonic phase [1]. The mandibular changes are often impressively developed distal to the row of replacement teeth [9], [14] and often show characteristic deformations of the mandibular angle, foramen, and ramus, as well as the mandibular processes [9]. Assessment of the influence of nerve damage on facial expression, soft and hard tissue changes should consider dysplastic changes of the trigeminal nerve (masticatory musculature, skeleton) in addition to the predominantly considered consequences of facial nerve damage [45], at least in patients with NF1.

## Conclusion

The examination of PA cephalograms of NF1 patients reveals topographical relationships of a NF1-characteristic tumor (i.e., the plexiform neurofibroma) and associated bone changes in the facial skeleton. Despite considerable individual variability in the mandibular findings, a pattern of bone changes characterizing the frame of the bone was proven based on cephalometric readings. Knowledge of this pattern is an important diagnostic background for the planning of reconstructive skeletal interventions, but also for assessing local bone changes in the course of the disease. In general, to distinguish between a tumor-related skeletal dysplasia and a tumor infiltration of the bone.

## Notes

### Competing interests

The authors declare that they have no competing interests.

### Authors’ contributions

REF and GC contributed equally to this publication.

Conceptualization of the study (REF, HAS), digitization and archiving of the measurement objects (GC), extension, testing, and validation of the cephalometric software (GC, HAS), cephalometric measurements (GC, HAS), evaluation of the data (GC, HAS, REF), drafting of the manuscript (REF, GC, HAS). Review and approval of the manuscript for publication: all authors.

### Acknowledgements

The authors would like to thank Computerforum, Elmshorn, Germany, for supporting the adaptation of the cephalometric software to the needs of this study. Many thanks to Ms. S. Wuttke, Photographic Department, UKE, for making the drawings. Special thanks to Prof. Hagel, Neuropathology, UKE, for expert histopathological diagnosis.

## Figures and Tables

**Table 1:**
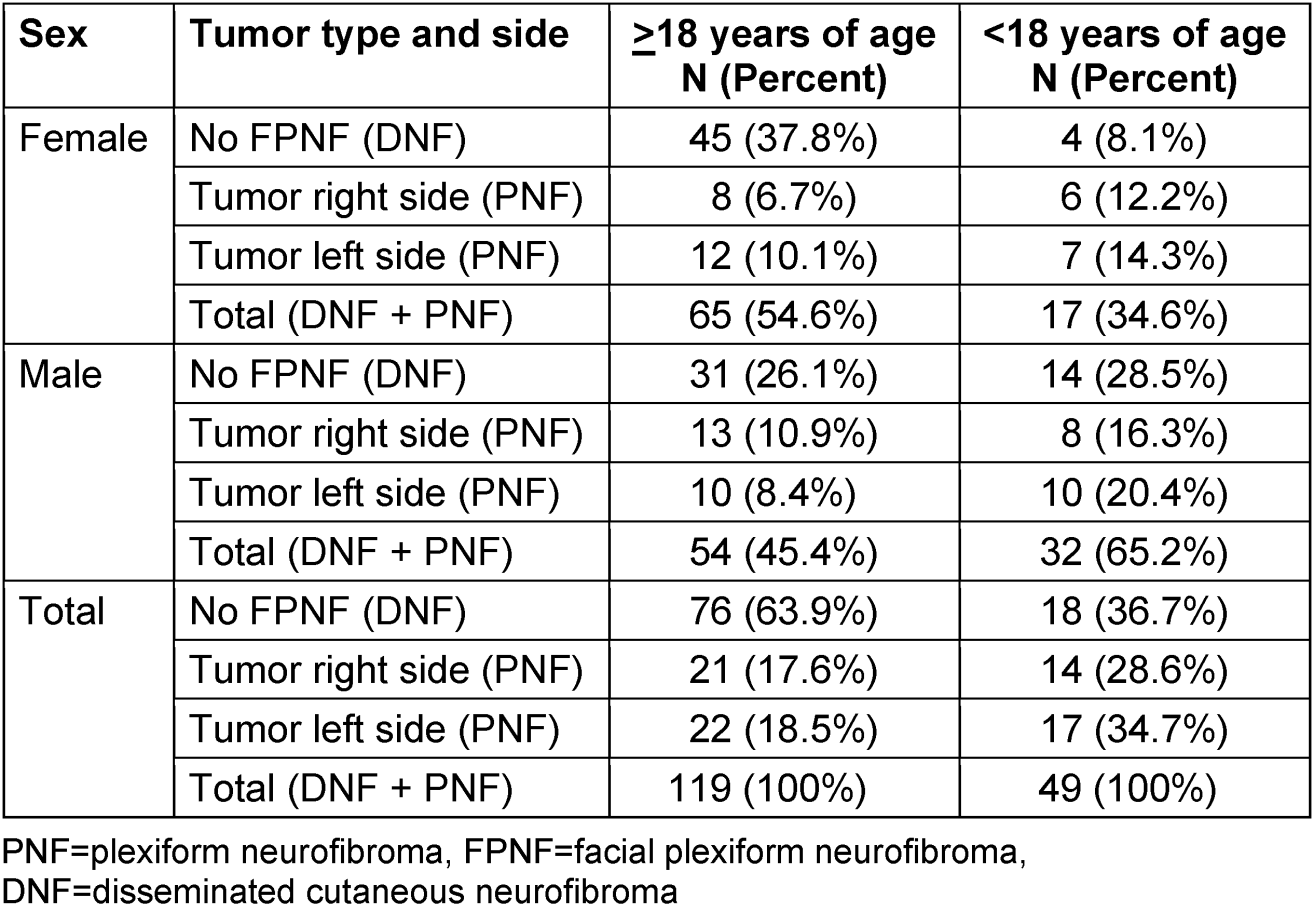
Number of patients with neurofibromatosis type 1 and side of the facial plexiform neurofibroma (PNF)

**Table 2:**
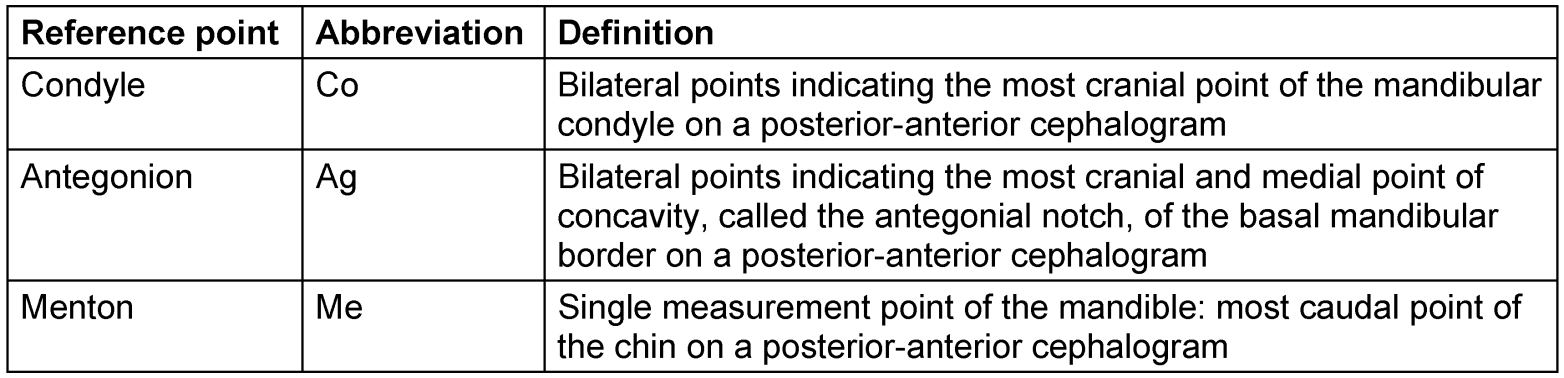
Definition of cephalometric landmarks

**Table 3:**
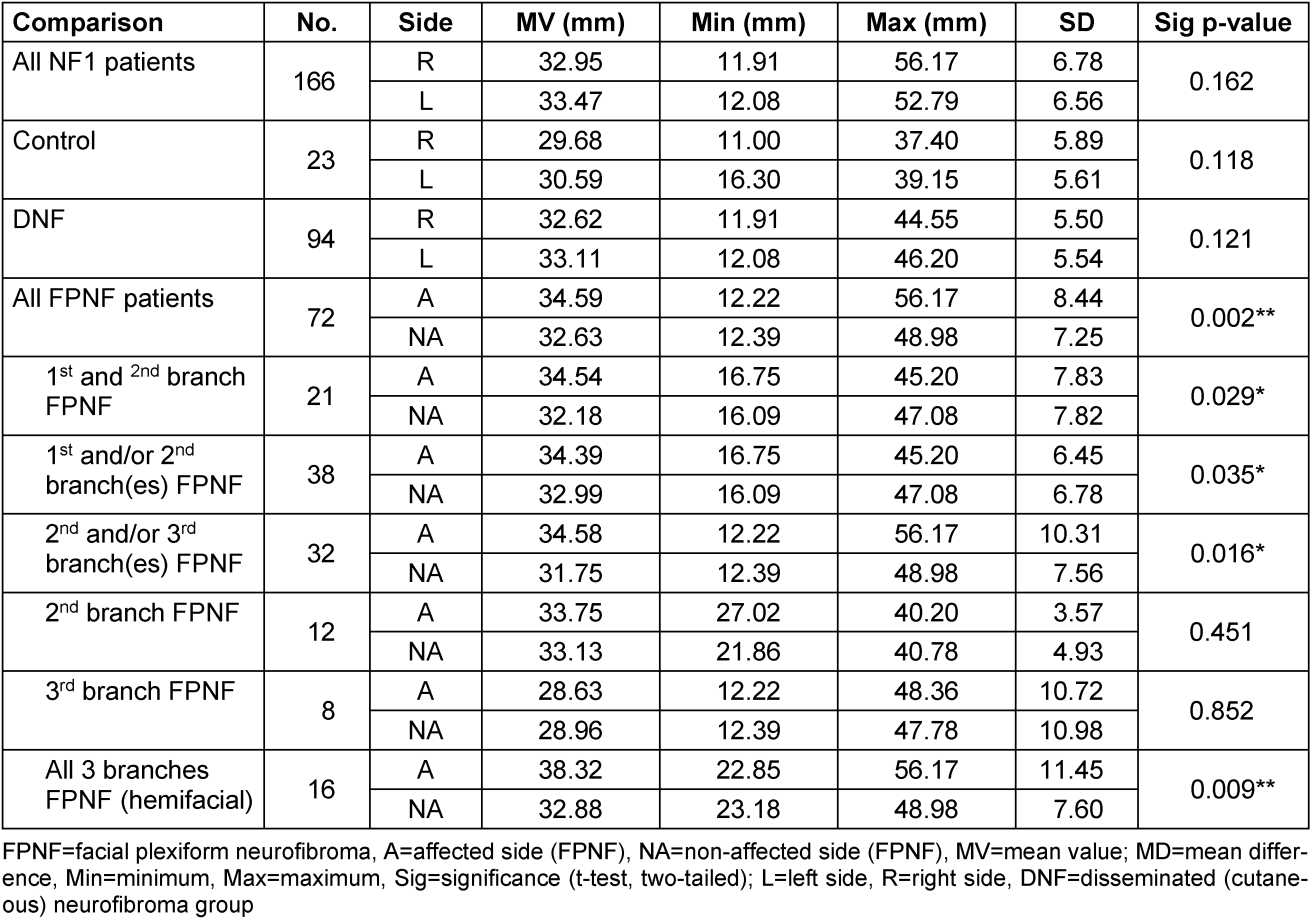
Vertical distance of reference points ‘Condyle’ to Z-plane on posterior-anterior cephalogram in NF1 patients (all ages) with facial plexiform neurofibroma (FPNF). Mean comparisons of the bilaterally registered measured values within the diagnostic groups. All FPNF manifestations are unilateral. The diagnostic group ‘FPNF’ is divided into subgroups according to the number of affected branches of the trigeminal nerve.

**Table 4:**
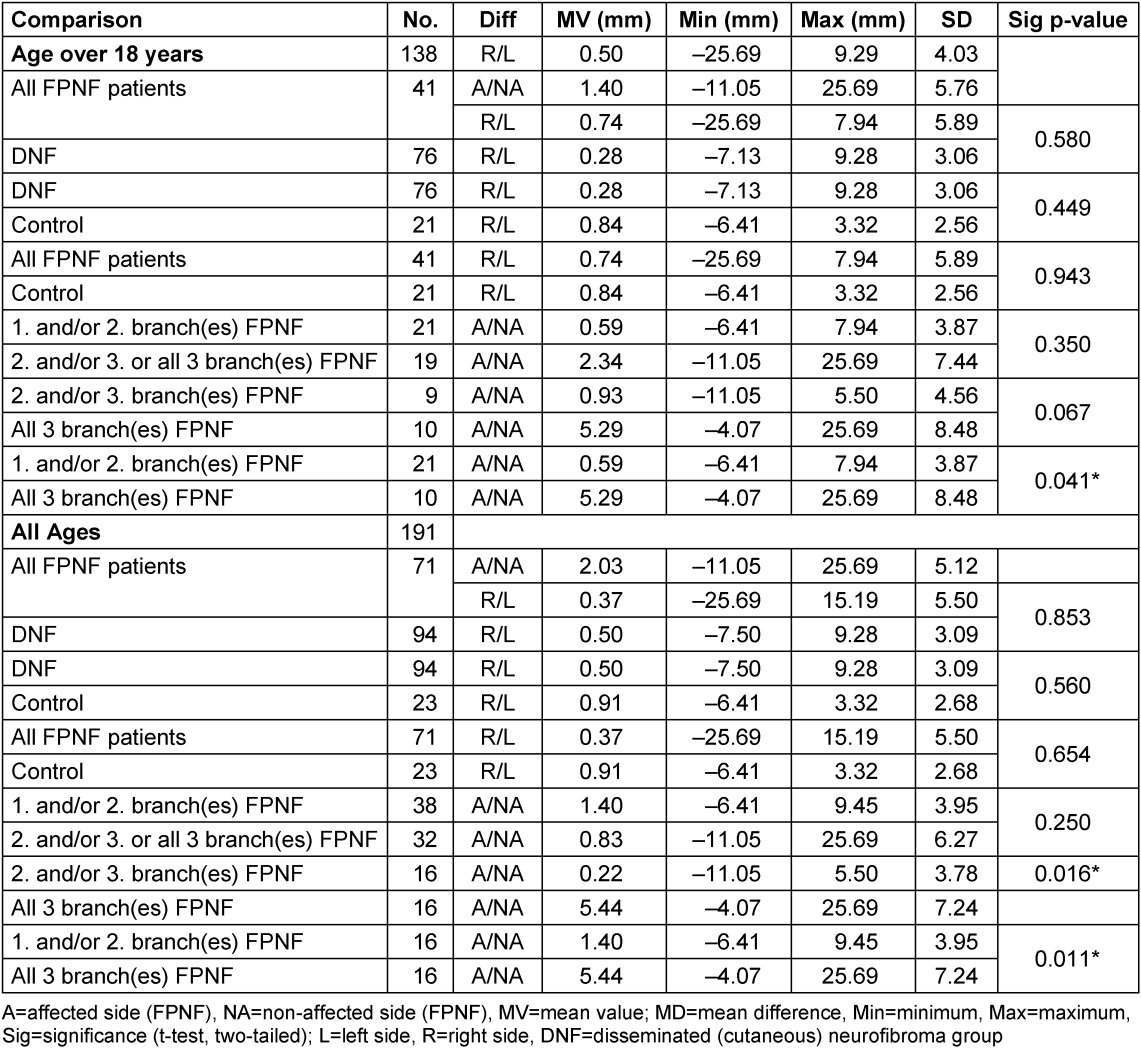
Comparison of individual vertical distance differences of reference points ‘Condyle’ to Z-plane (CoR-Z minus CoL-Z) on posterior-anterior cephalogram in NF1 patients with facial plexiform neurofibroma (FPNF). All tumor manifestations are unilateral. The diagnostic group is divided into subgroups according to the location of affected branches of the trigeminal nerve.

**Table 5:**
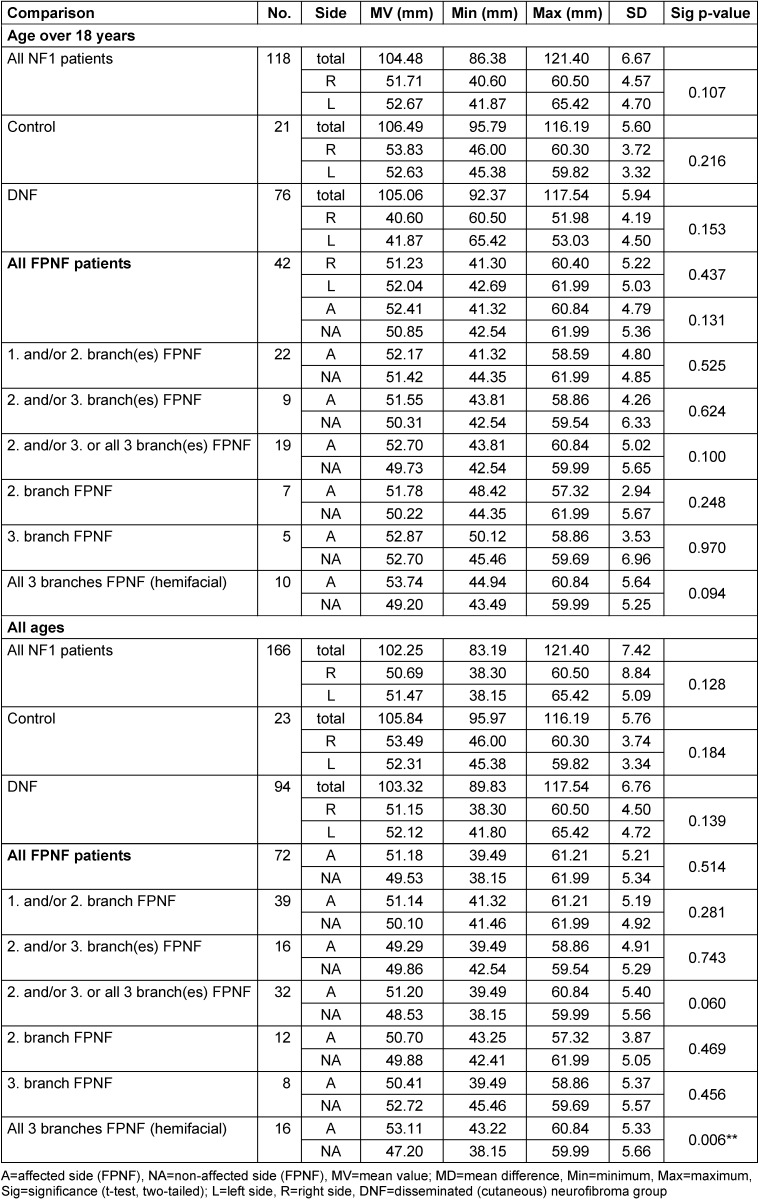
Horizontal distance of reference points ‘Condyle’ to M-plane on posterior-anterior cephalogram in NF1 patients with facial plexiform neurofibroma (FPNF): Intraindividual comparisons. All tumor manifestations are unilateral. The diagnostic group is divided into subgroups according to the location of affected branches of the trigeminal nerve.

**Table 6:**
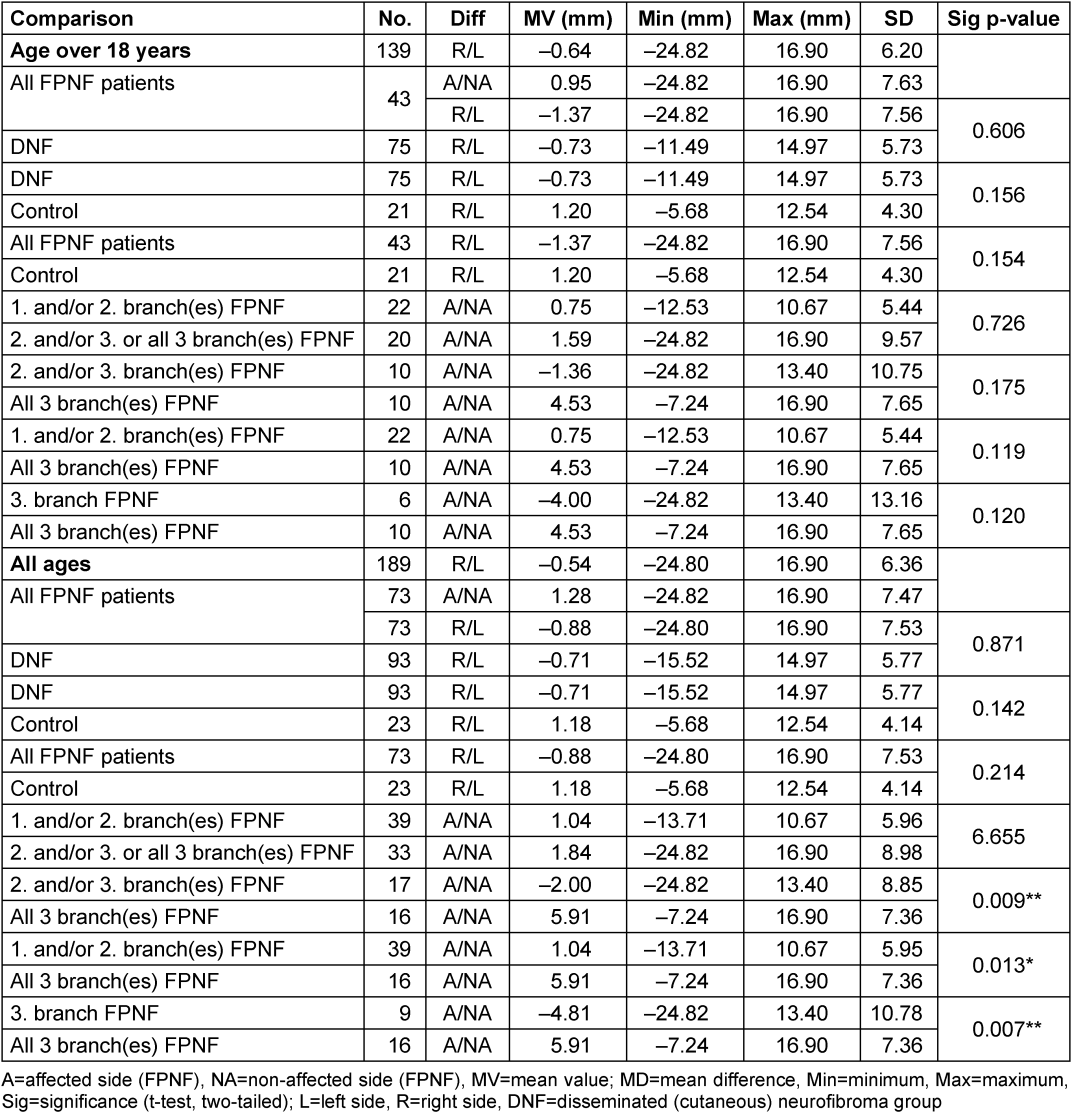
Comparison of individual horizontal distance differences of reference points ‘Condyle’ to M-plane (Co-MR minus Co-ML; CoA-M minus CoNA-M) on PA cephalograms in NF1 patients with facial plexiform neurofibroma (FPNF). All tumor manifestations are unilateral. The diagnostic group is divided into subgroups according to the location of affected branches of the trigeminal nerve. For the interpretation of the measured values, it is important to consider following definitions: Difference R_L has a positive value: The right condyle is farther away from the M-plane than the left condyle. Difference R_L has a negative value: The left condyle is farther away from the M-plane than the right condyle. Right/left distinction was used for measurements of the DNF group. Difference A_NA is positive: The measuring point of side A (affected side) is farther away from the M-plane than the measuring point of side NA (non-affected). Difference A_NA is negative: The measuring point of side A (affected side) is closer to the M-plane than the measuring point of side NA (non-affected). The A/NA distinction was used for measurements of the FPNF group.

**Table 7:**
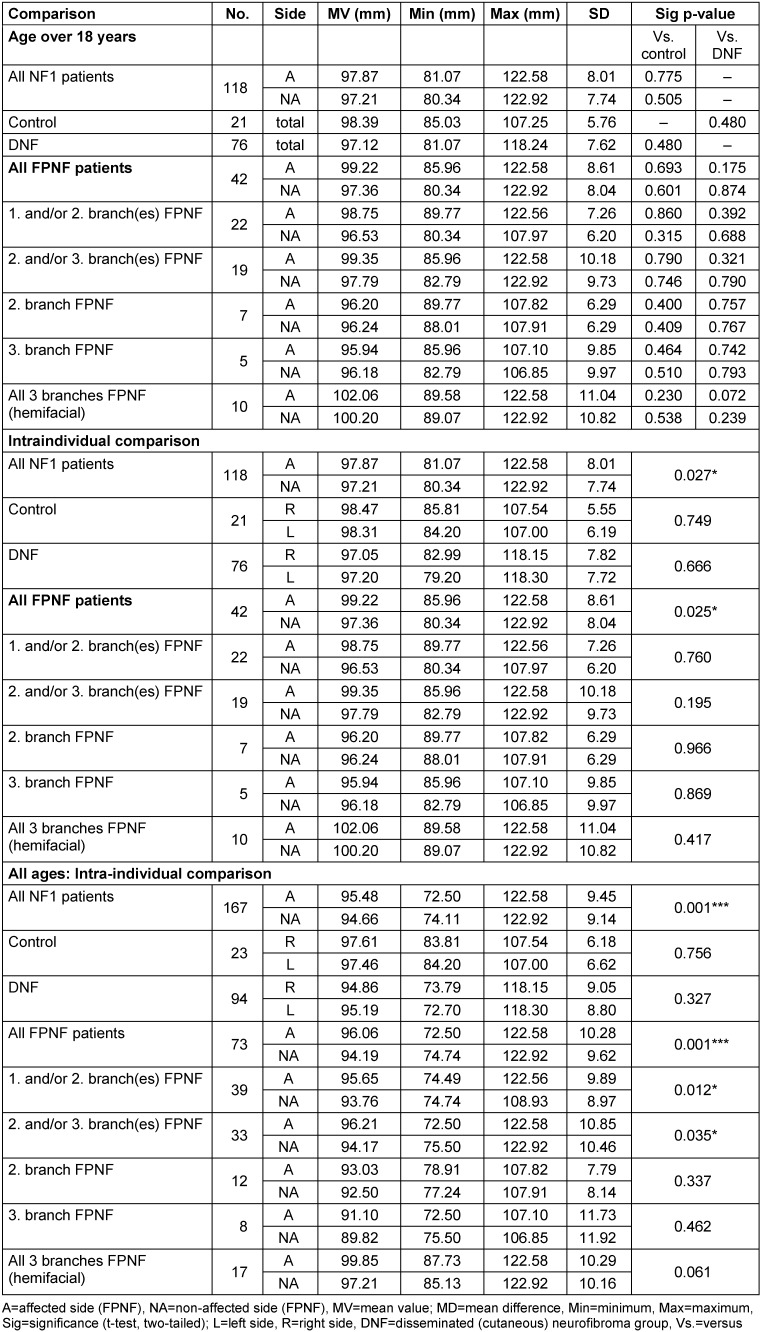
Vertical distances of reference points ‘Antegonion’ to Z-plane on posterior-anterior cephalograms in NF1 patients with facial plexiform neurofibroma (FPNF): intraindividual differences. All FPNF manifestations are unilateral. The diagnostic group is divided into subgroups according to the number of affected branches of the trigeminal nerve.

**Table 8:**
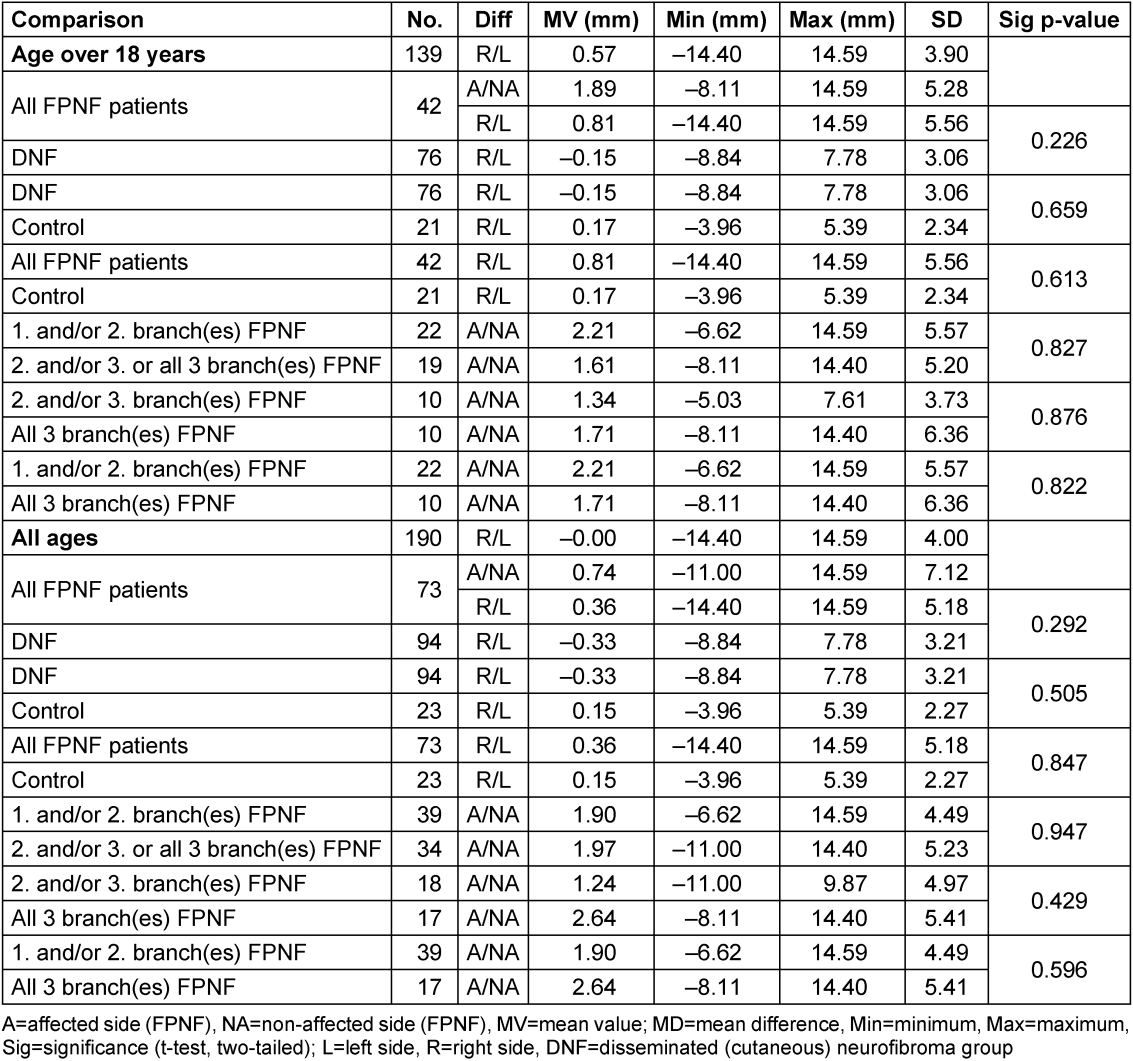
Comparison of individual vertical distance differences of reference points ‘Antegonion’ to Z-plane on posterior-anterior cephalogram in NF1 patients with facial plexiform neurofibroma (FPNF): group comparisons. All FPNF manifestations are unilateral. The diagnostic group is divided into subgroups according to the location of affected branches of the trigeminal nerve.

**Table 9:**
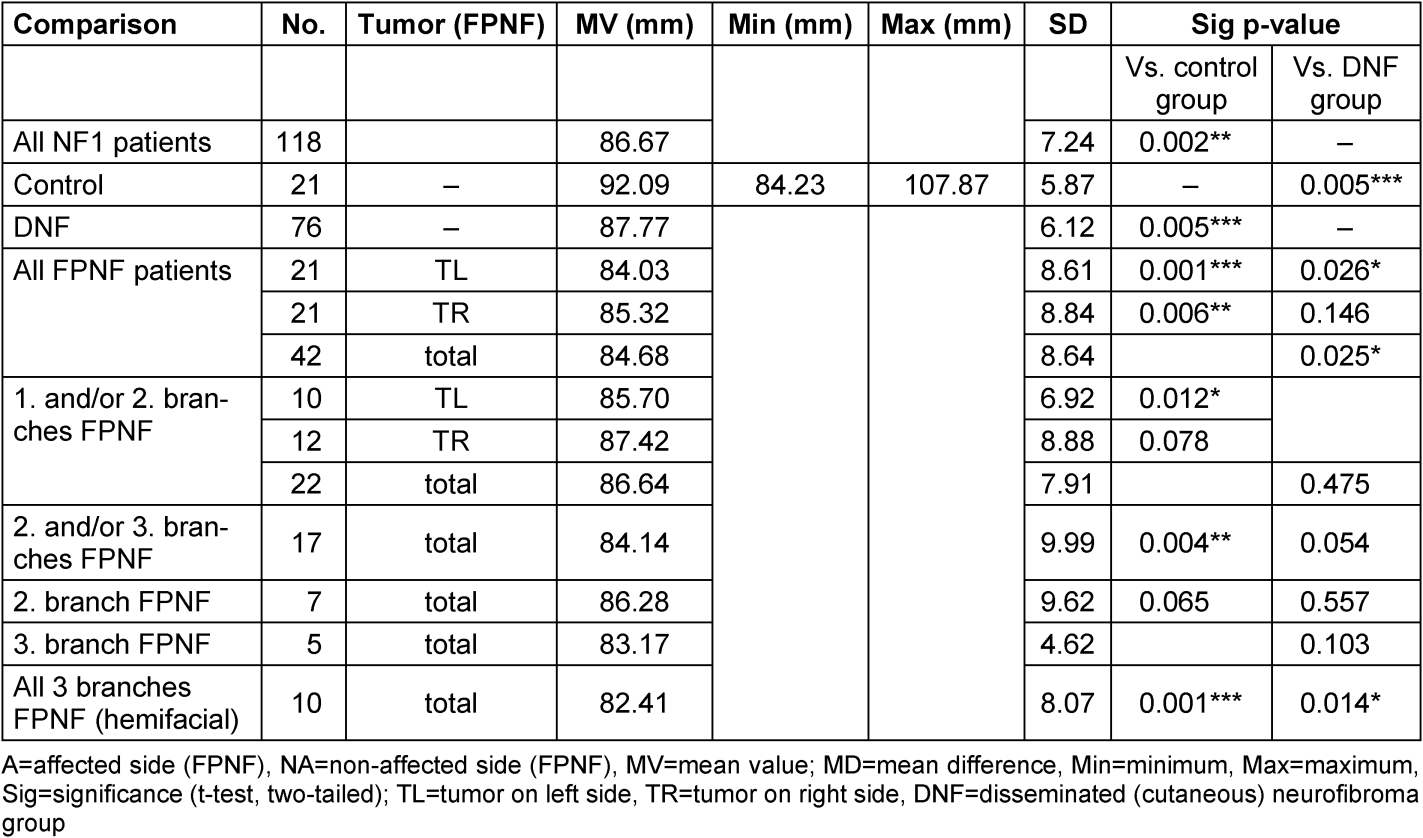
Total horizontal distance of bilateral reference point ‘Antegonion’ (AgR-AgL) on posterior-anterior cephalogram in NF1 patients with facial plexiform neurofibroma (FPNF) (≥18 ys). All tumor manifestations are unilateral. The diagnostic group is divided into subgroups according to the number of affected branches of the trigeminal nerve.

**Table 10:**
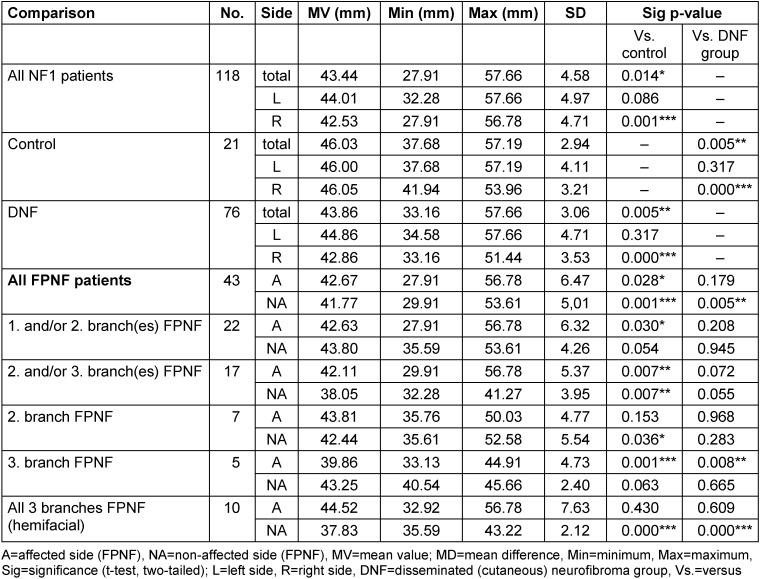
Horizontal distance of bilateral reference point ‘Antegonion’ to M-plane on posterior-anterior cephalogram in NF1 patients with facial plexiform neurofibroma (FPNF) (≥18 ys): Comparison with the control group and DNF group. All tumor manifestations are unilateral. The diagnostic group is divided into subgroups according to the number of affected branches of the trigeminal nerve.

**Table 11:**
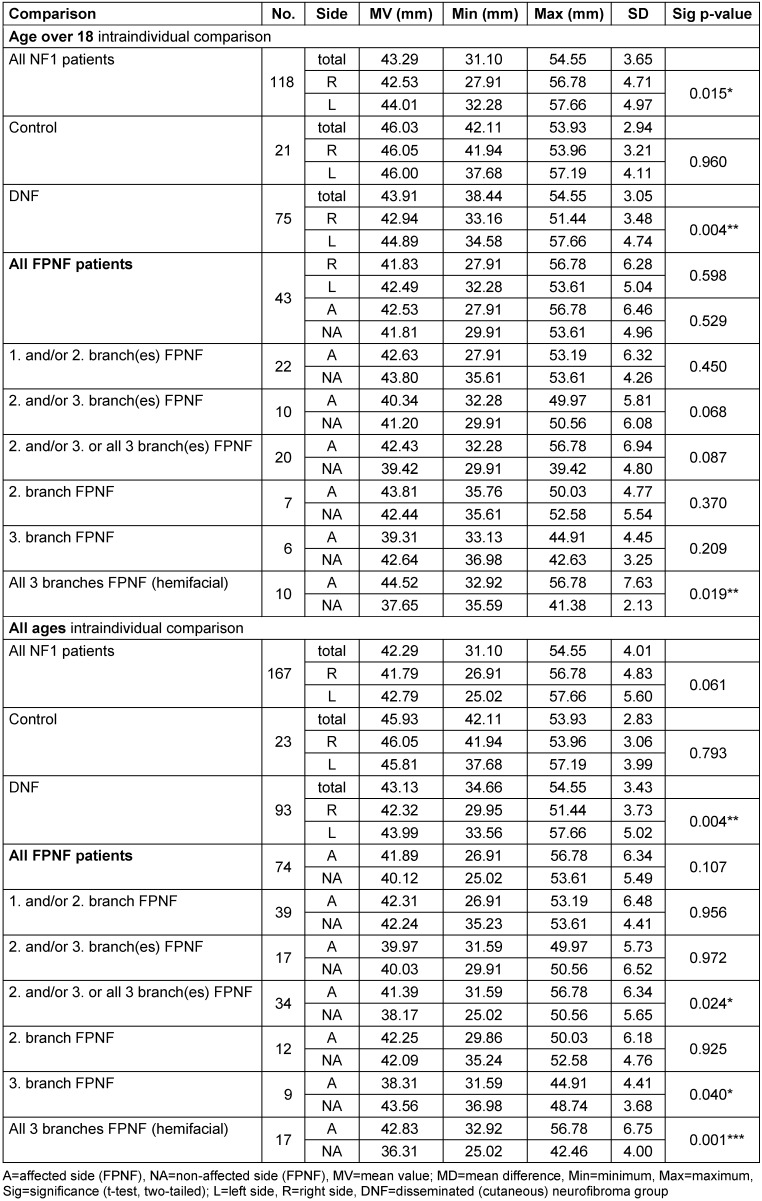
Horizontal distance of reference points ‘Antegonion’ to M-plane on posterior-anterior cephalogram in NF1 patients with facial plexiform neurofibroma (FPNF): Intraindividual comparisons. All tumor manifestations are unilateral. The diagnostic group is divided into subgroups according to the number of affected branches of the trigeminal nerve.

**Table 12:**
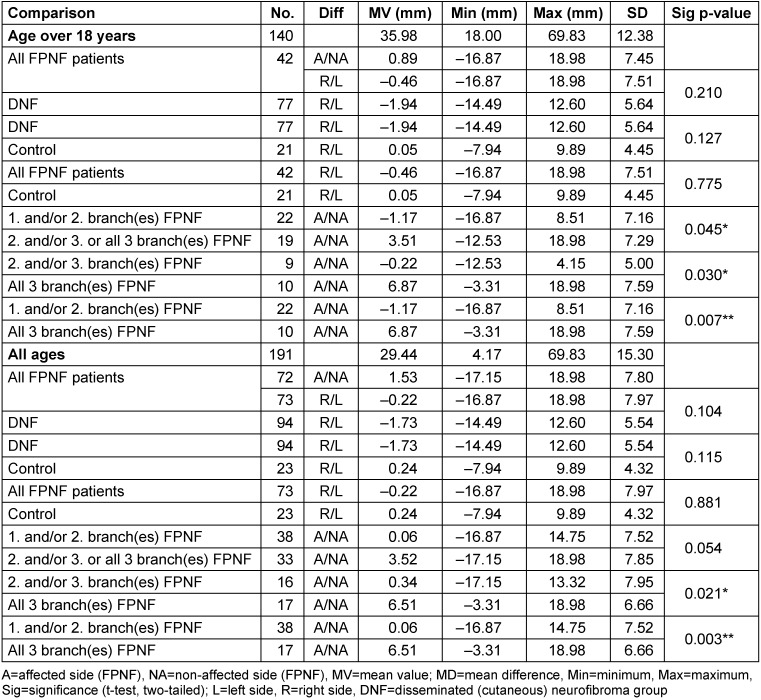
Table 12: Comparison of horizontal distance differences of reference points ‘Antegonion’ to M-plane (AgR-M minus AgL-M) on PA cephalogram in NF1 patients with facial plexiform neurofibroma (FPNF). All tumor manifestations are unilateral. The diagnostic group is divided into subgroups according to the location of affected branches of the trigeminal nerve. A/NA positive: Tumor side larger; A/NA negative: Tumor side smaller; R/L positive: Right side larger; R/L negative: Left side larger.

**Table 13:**
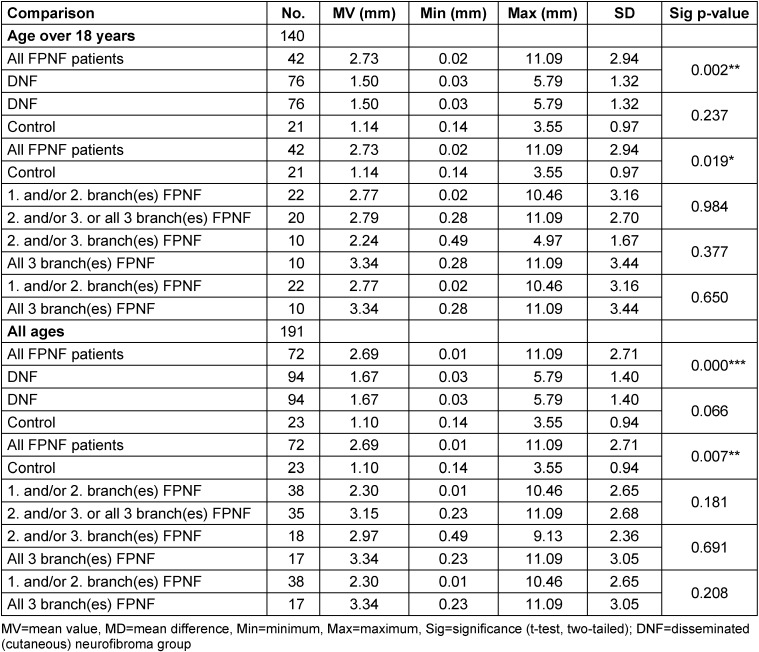
Angle between lines ‘Antegonion’ to Z-plane on posterior-anterior cephalogram in NF1 patients with facial plexiform neurofibroma (FPNF). All tumor manifestations are unilateral. The diagnostic group is divided into subgroups according to the location of affected branches of the trigeminal nerve.

**Table 14:**
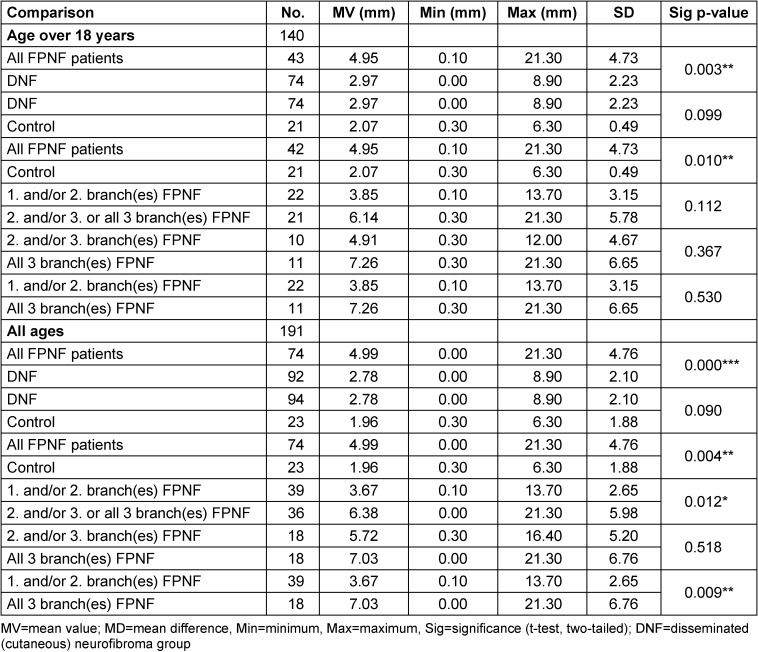
Distance ‘Menton’ to M-plane on posterior-anterior cephalogram in NF1 patients with facial plexiform neurofibroma (FPNF). All tumor manifestations are unilateral. The diagnostic group is divided into subgroups according to the location of affected branches of the trigeminal nerve.
